# Zooplankton mortality and distribution around a seismic survey

**DOI:** 10.1038/s41598-025-09465-2

**Published:** 2025-09-30

**Authors:** Emilie Hernes Vereide, Anne Christine Utne-Palm, Josefin Titelman, Geir Pedersen, Espen Strand, Marina Mihaljevic, Saskia Kühn, Dag Altin, Anders Thorsen, Lucie Campillo, David M. Fields, Babak Khodabandeloo, Karen de Jong

**Affiliations:** 1 Ecosystem Acoustics Group, Institute of Marine Research, Nykirkekaien 1, Bergen, NO-5004 Norway; 2 Fish Capture Group, Institute of Marine Research, Nykirkekaien 1, Bergen, NO-5004 Norway; 3 Department of Biosciences, University of Oslo, PO Box 1066 Blindern, Oslo, NO-0316 Norway; 4 Plankton Group, Institute of Marine Research, Nordnesgaten 50, Bergen, NO-5005 Norway; 5 Austevoll Research Station, Institute of Marine Research, Sauganeset 16, Storebø, NO-5392 Norway; 6 Coastal Ecology, Kiel University, Research and Technology Centre West Coast, Hafentörn 1, 25761 Büsum, Germany; 7 BioTrix, Trondheim, NO-7022 Norway; 8 Research Infrastructure SeaLab, Faculty of Natural Sciences, Norwegian University of Science and Technology, Trondheim, NO-7491 Norway; 9 Reproduction and Developmental Biology, Institute of Marine Research, Nordnesgaten 50, Bergen, NO-5005 Norway; 10 Bigelow Laboratory for Ocean Sciences, East Boothbay, ME 04544 USA

**Keywords:** Impulsive underwater noise, Seismic survey, Copepod, Zooplankton, Mortality, Vertical distribution, Marine biology, Environmental impact

## Abstract

Anthropogenic underwater noise from seismic airguns is increasing, posing a potential threat to marine life. Despite their ecological importance, knowledge of the impacts of seismic surveys on zooplankton remains limited. In the North Sea, we utilized various methods to assess potential effects on zooplankton around an ongoing seismic survey. A seismic airgun array (3060 inch^3^) performed a survey along parallel lines, while we sampled from a research vessel positioned at the end of three of these lines. The airgun-generated noise reached a maximum broadband sound exposure level of 182 dB re 1µPa^2^s at the closest proximity (~50 m). Zooplankton biomass exhibited a consistent distribution in line with hydrography and chlorophyll distribution, before and after airgun exposure. Immediate mortality in *Calanus* spp. was uniform across sound levels, never exceeding 35.9%. Cultured *Calanus finmarchicus* were exposed while submerged in bags. These displayed low immediate mortality (<10%), with an increasing trend (<30%) up to 7 days post-exposure. These findings highlight the interplay between sound exposure, environmental conditions, and the impact on zooplankton in areas of seismic activity, indicating that immediate impacts of seismic surveys on zooplankton may be limited. However, delayed impacts that could lead to population effects require further investigation.

**Supplementary Information:**

The online version contains supplementary material available at 10.1038/s41598-025-09465-2.

## Introduction

The impact of anthropogenic underwater noise on marine life has emerged as an increasing cause of concern^1,2^. Anthropogenic underwater noise is comprised of diverse sources, encompassing continuous noise such as boat traffic and operational wind turbines, as well as impulsive noise from activities such as pile driving, underwater explosions, and seismic surveys using airguns that produce loud sounds to map the sea bottom^3^. While the effects of seismic surveys on marine mammals and fish, such as acoustic masking, hearing loss, behavioral changes, and physical damage, are relatively well-explored^4–10^, our understanding of their impact on zooplankton is limited^11^.

Seismic surveys, involving the use of seismic airgun arrays, are globally employed for oil and gas exploration and seafloor mapping, often spanning weeks or months^6^. On the Norwegian Shelf, seismic exploration began in the late 1960s and now covers an area of approximately 20 000 km^2^ annually (as of 2021)^12^. During these surveys, arrays of airguns, typically organized in sets of 18 to 48 airguns, generate acoustic waves through the sudden release of high-pressure air stored in the airguns. The energy of the generated sound is mainly concentrated within low frequencies (below ~100 Hz). Although the sound pressure amplitude decreases with distance, the lower frequencies can propagate over hundreds to thousands of kilometers from the source because they are less absorbed than higher frequencies^13,14^. Given the extensive and ongoing utilization of seismic surveys in diverse exploration activities, it is essential to enhance our understanding of the potential impacts of these surveys on marine life.

Zooplankton play a crucial role in pelagic food webs and contribute to the biological pump^15^. In the North Atlantic Ocean, the large (2–4 mm) copepod *Calanus* spp. is pivotal in the food web, serving as the primary food for many commercially important fish species, such as mackerel (*Scomber scombrus*), herring (*Clupea harengus*) and blue whiting (*Micromesistius poutassou*)^16,17^. The consequences of seismic surveys on zooplankton are not yet fully understood, but potential impacts include physical harm caused by fluid motion^18^, disorientation due to effects on their sensory systems^19^ or altered behavior due to rapid pressure changes related to airguns^20^.

Despite the ecological importance of zooplankton, knowledge on the effects of seismic surveys is scarce, with existing studies presenting inconsistent results^11,21,22^. For example, McCauley et al. (2017) observed a ~2.5-fold increase in dead zooplankton following exposure to a relatively small airgun (150 inch^3^) up to 1 km away from the source^19^. In contrast, Parry et al. (2002) found no discernible effects on bivalve larvae mortality after exposure to a full airgun array (3542 inch^3^)^23^. Mortality caused by the propeller of the sampling vessel were not controlled for in either of these studies. Fields et al. (2019), using a different approach without a vessel and exposing zooplankton in bags, reported lower mortality (max. 15% in exposed vs. ~2% in control) in the large copepod *C. finmarchicus*, but only at close proximity (<5 m) to two slightly larger airguns (each 260 inch^3^) than those used in McCauley et al. (2017)^18,19^. Similarly, Vereide et al. (2023) observed higher but limited mortality (14% in exposed vs. 4% in controls) in *Acartia tonsa* nauplii exposed to an array of two small airguns (each 40 inch^3^)^24^, and Pearson et al. (1994) reported no mortality in *Cancer magister* larvae exposed to seven airguns (total 840 inch^3^)^25^. Overall, studies investigating zooplankton mortality following seismic exposure demonstrate diverse outcomes influenced by various airgun sources and distances from the source.

To the best of our knowledge, this study is the first to investigate the effects of a seismic survey on zooplankton mortality and distribution across varying distances and nearby (<50 m) an ongoing seismic survey using a full airgun array. Most experiments exploring the impact of anthropogenic noise on invertebrates have been conducted in the laboratory^11,26^. While laboratory experiments contribute valuable insights into the mechanisms involved, large scale field experiments near actual seismic surveys (this study) can provide direct evidence of the effects of real noise sources in a natural environment^21,27,28^.

To assess the influence of seismic surveys on zooplankton mortality and distribution at various distances from the seismic source, and to characterize the sound levels generated by the seismic airgun array, we utilized a diverse range of experimental methods and field sampling techniques. Our approach encompassed investigations into both natural zooplankton populations and cultured copepods, providing a comprehensive understanding of the potential impacts of seismic surveys on zooplankton.

## Results

### Sound levels

**Fig. 1:**
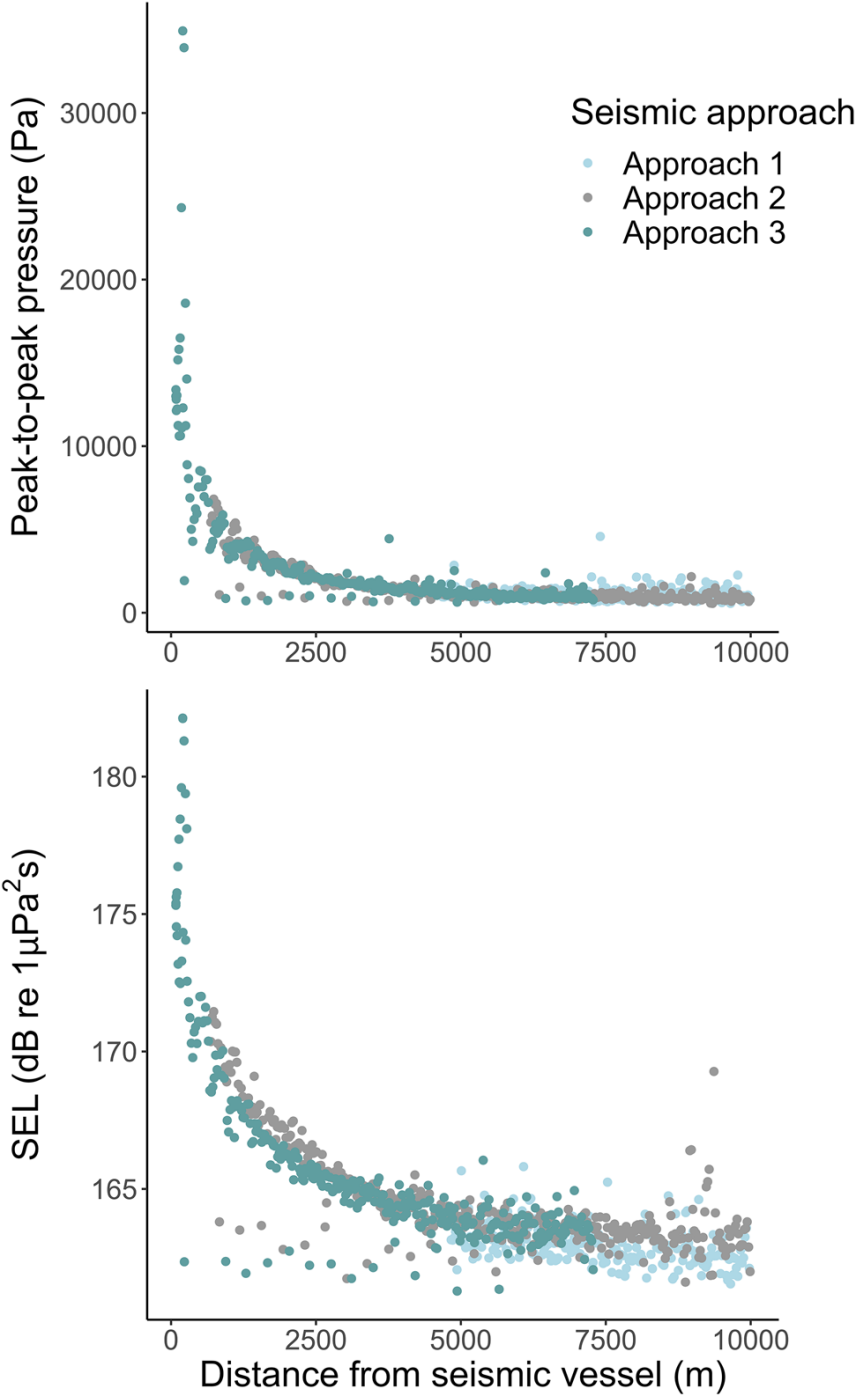
Pressure and sound levels from seismic approach 1–3 recorded as peak-to-peak pressure (Pa) (upper panel), and broadband sound exposure level (SEL), over 10 Hz to 20 kHz (dB re 1µPa^2^s) (lower panel).

Sound levels were measured with a hydrophone deployed from the research vessel during all three seismic approaches. From a distance of approximately 6 km from the seismic source, airgun-generated sound exceeded the noise from the research vessel of 158 ± 0.5 dB re 1 µPa² s (broadband SEL; Fig. 1). The sound exposure level showed a consistent relation with distance across all three seismic approaches (1–3), reaching its maximum output of 182 dB re 1 µPa² s in the broadband spectrum closest to the hydrophone (seismic approach 3). For the peak-to-peak pressure levels (Pa), the maximum was 35 kPa (seismic approach 3; Fig. 1).

### Bottom-mounted echosounders (WBAT)—zooplankton vertical movement

**Fig. 2:**
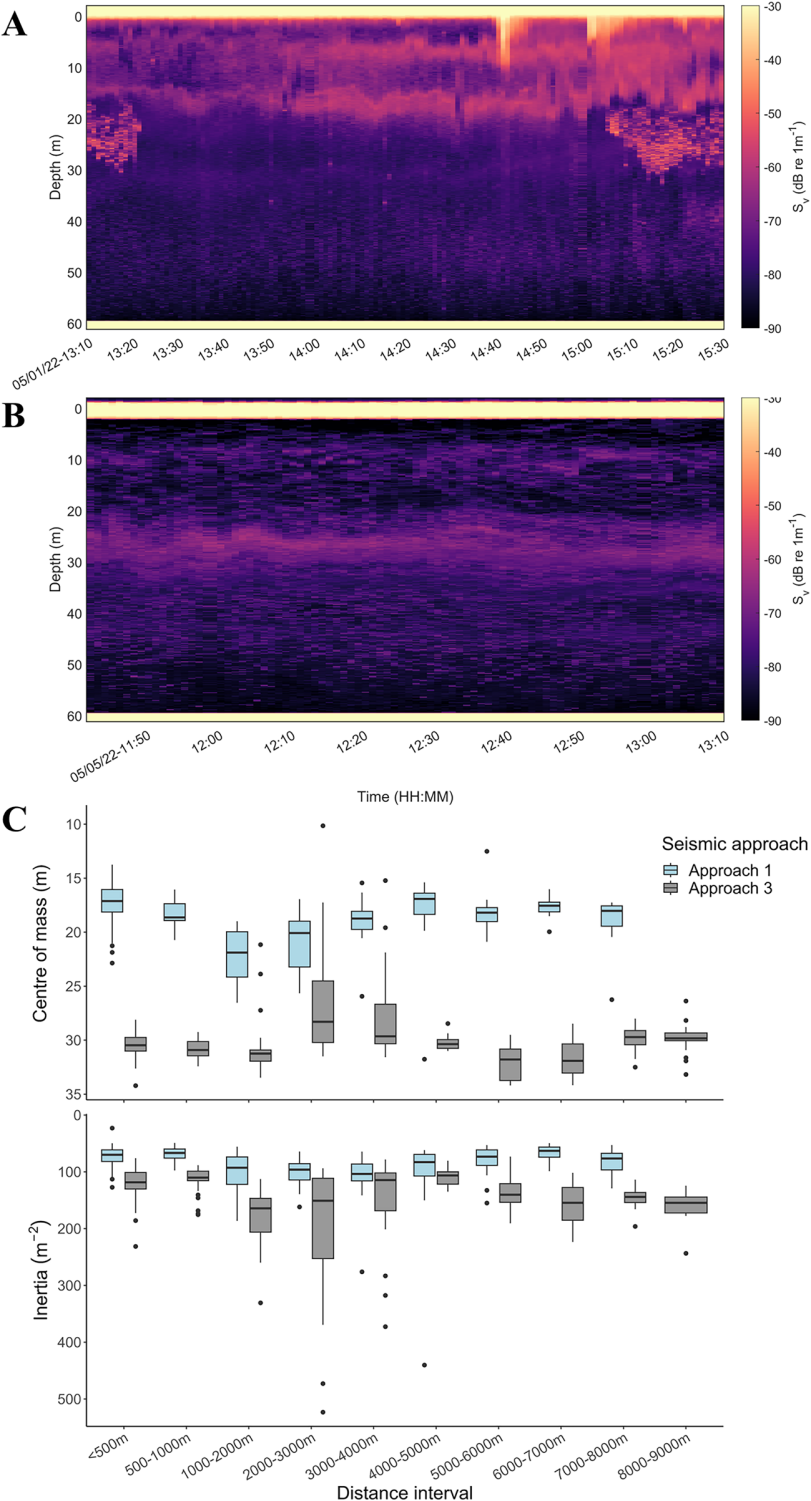
Overview of the WBAT data from the seismic approaches that was included in the analysis (1, 3) during exposure. **A** Approach 1 (1st of May), **B** Approach 3 (5th of May), **C**), Centre of mass (CM; m) and inertia (I; m^− 2^) during seismic approach 1 and 3 for distance intervals up to 9 km away from the seismic source. All echograms from the specific exposure dates from each WBAT can be found in Supplementary Material.

Zooplankton vertical movement was recorded using acoustic data from three bottom-mounted, upward-facing echosounders positioned at fixed locations during the seismic approaches. The dispersion of zooplankton (inertia; I) increased with distance from the seismic airguns for the two approaches included in the analyses (GLM, Approach 1; *p* = 0.008, Approach 3; *p* <0.001; Fig. 2). In contrast, there was no significant effect of the decreasing distance with the seismic source on the center of mass of zooplankton along the entire seismic approach (GLM, Approach 1; *p* = 0.178, Approach 3; *p* = 0.603; Fig. 2), indicating no or limited changes in vertical movement in zooplankton due to seismic shooting. The variability between the seismic approaches exceeded the fluctuations observed along a single seismic approach (Fig. 2) but was smaller than the changes in distribution governed by the diel vertical migration of the dominating zooplankton.

### Zooplankton biomass—vertical distribution

**Fig. 3:**
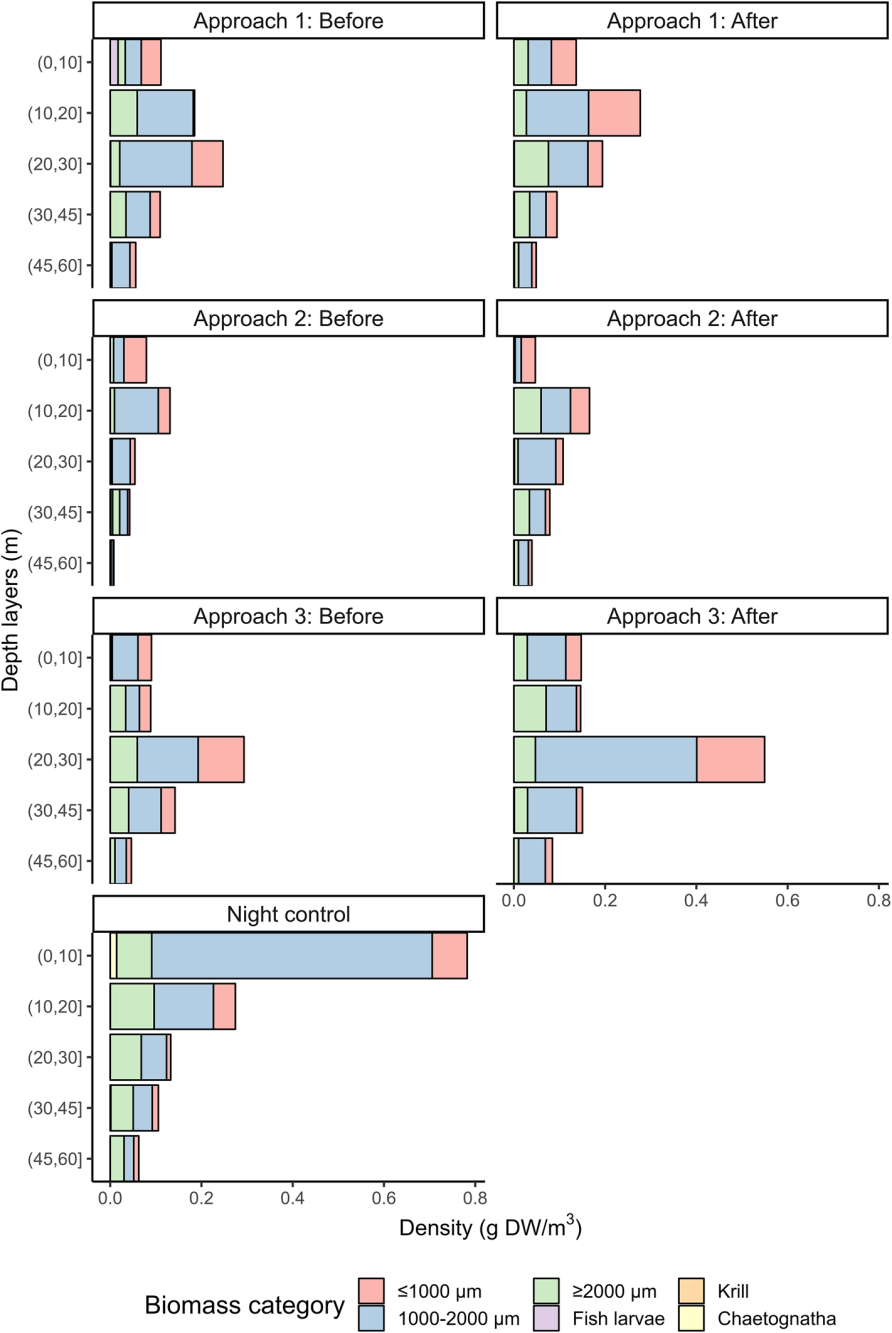
Zooplankton biomass (g DW m^− 3^) within different size fractions (≤ 1000 μm, 1000–2000 μm, ≥ 2000 μm) and animal categories; fish, krill, and chaetognaths, for depth intervals 0–10 m, 10–20 m, 20–30 m, 30–45 m, 45–60 m. A complete overview of sampled taxa from seismic approach 1 (before), seismic approach 3, and night control can be found in Supplementary Material.

The vertical distribution of zooplankton biomass was assessed with a Multinet, which sampled zooplankton at discrete depth intervals at the start and end of each seismic approach. The 1000–2000 μm size fraction, comprised primarily of *C. finmarchicus* (~90%) and *Calanus helgolandicus* (~10%), dominated the zooplankton biomass across the various depth intervals and stations (Fig. 3). During daytime, much of the zooplankton was distributed around the deep chlorophyll-*a* maximum (Fig. 3, see Supplementary Material for environmental conditions). Overall, the zooplankton vertical distribution appeared relatively similar before and after seismic shooting activity (Fig. 3). However, in seismic approach 1, the biomass peak shifted from 20–30 m depth to 10–20 m depth post-shooting. The peak abundance of zooplankton was deeper (20–30 m) in seismic approach 3 compared to 1 and 2, in correspondence with a wind-induced deepening of the distribution of chlorophyll-*a*. During the night (control), zooplankton migrated upwards, with a biomass peak of 0.8 g DW m^− 3^ in the upper 10 m (Fig. 3).

In terms of abundance, small echinoderm larvae dominated across all samples in the surface layers. A comprehensive overview of all represented taxa and the distribution of *Calanus* spp. developmental stages from seismic approach 1 and 3 is given in Supplementary Material.

### Immediate mortality of field caught *Calanus* spp.

**Fig. 4:**
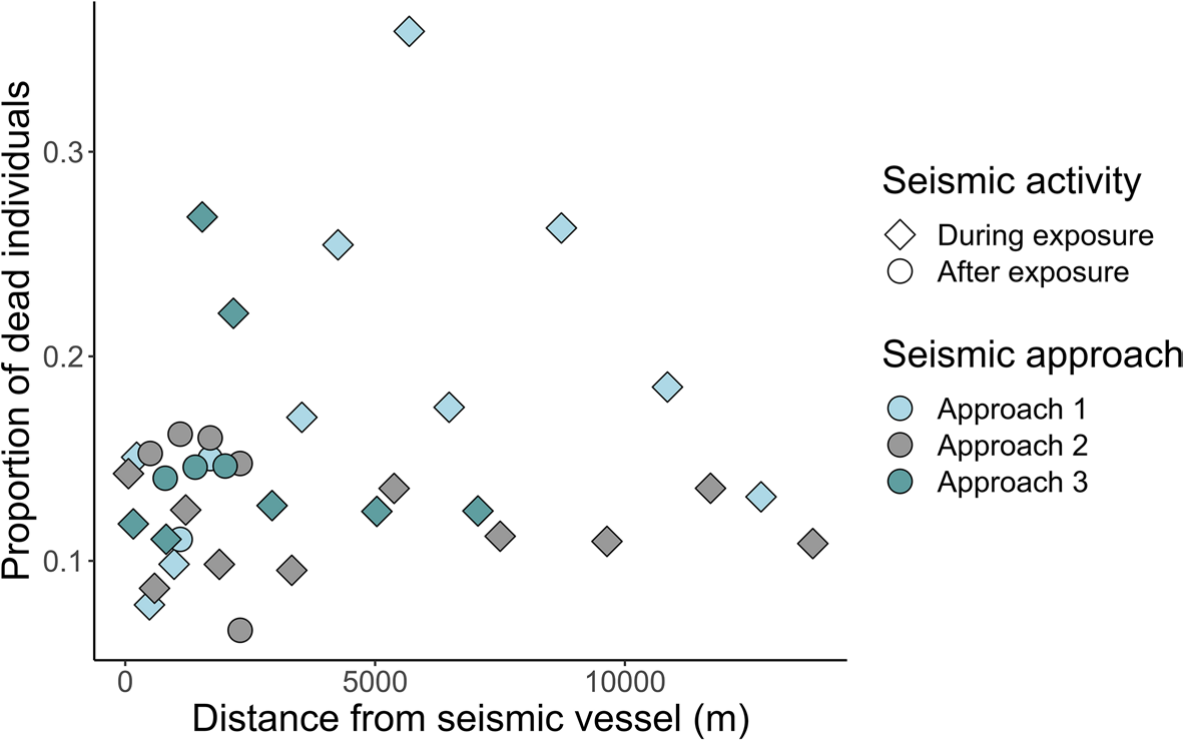
Mortality in large copepods from seismic approach 1–3, sampled at different distances from the seismic source. The squared shapes were sampled during shooting as the seismic vessel was approaching, and the circular shapes were sampled after the seismic vessel had passed the research vessel and stopped shooting.

To assess the immediate mortality of *in situ* zooplankton, WP2 nets were deployed multiple times during each seismic approach, and the proportion of live zooplankton was determined using Neutral Red Stain. The highest recorded mortality occurred at a distance of 6 km from the seismic vessel (SEL ~163 dB re 1 µPa^2^ s) where 35.9% of the copepods were dead. Aside from this particular instance, the mortality fraction consistently remained below 30% (Fig. 4), with no consistent patterns along the transects. In other words, there was no significant correlation between the immediate mortality of field caught *Calanus* spp. and distance from the seismic source during exposure (Spearman correlation test, n_WP2 nets_=27, *p* = 0.27) (Fig. 4). Additionally, there was no difference in mortality between *Calanus* spp. sampled during and after exposure (Mann Whitney, n_WP2 nets_=37, *p* = 0.56).

### Immediate and delayed mortality of cultured *Calanus*

The immediate and delayed mortality (up to 7 days after exposure) were measured in cultured *C. finmarchicus* using two main approaches; (i) with bags of 10 individuals introduced at different distances to the seismic airgun source during all three seismic approaches, and (ii) with bags containing 50 individuals introduced close to the seismic airgun source during one seismic approach.

**Fig. 5:**
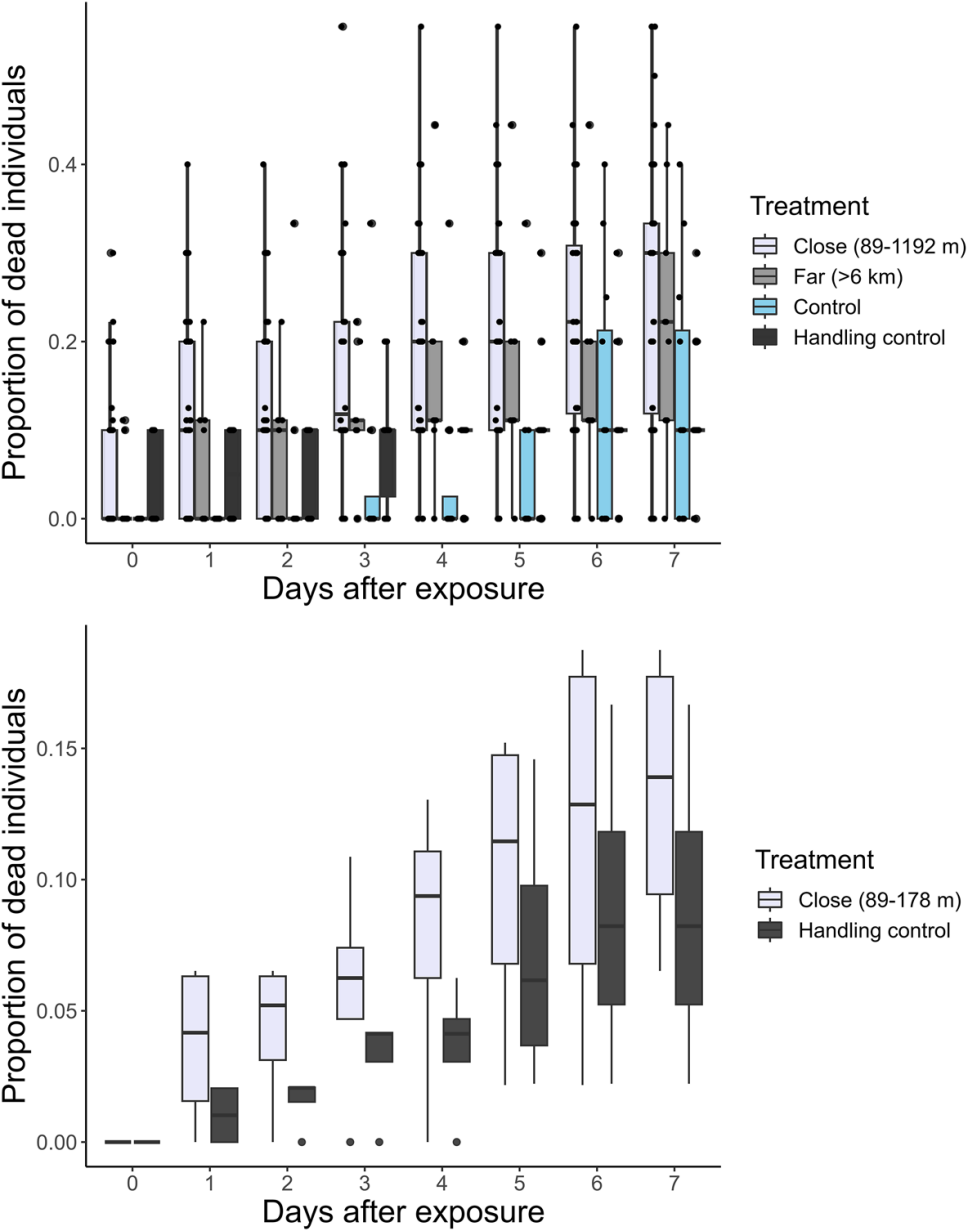
The proportion of dead individuals (*C. finmarchicus*) immediately after treatment (day 0) and up to 7 days after. Upper panel) Bags of 10 individuals (exposure from close distance; 89–1192 m from the seismic vessel; *n* = 36, exposure from far distance; >6 km from the seismic vessel; *n* = 9, normal control; *n* = 12, and handling control; *n* = 18. Lower panel) Bags of 50 individuals (exposure from close distance; 89–178 m from the seismic vessel; *n* = 4, and handling control; *n* = 4.

In the bags with 10 individuals, the cumulative mortality in the bags exposed from close distance was significantly higher than the controls (GLM, n_bags_=12 (control), 18 (handling control), 9 (far exposure), 36 (close exposure), *p* = 0.007; Fig. 5). After 7 days, the mortality was 25.1 ± 15.3% (mean ± SD) in the airgun exposure from close distance, 22.3 ± 14.2% from far distance (>6 km), and 12.7 ± 14.2% and the 11.7 ± 7.1% in the regular and handling controls. However, the immediate mortality was not significantly different from the controls at close (5.4 ± 8.2%) or far (2.3 ± 4.7%) distance from the seismic source.

In the bags with 50 individuals, the cumulative mortality in the airgun exposed *C. finmarchicus* from a close distance (89–178 m) was lower than in the bags with 10 individuals from close distance (89–1192 m). In the bags with 50 individuals, there were no dead individuals in either the airgun exposed treatment (89–178 m from the airgun source) or the handling control immediately after treatment (Fig. 5). There was no significant effect on the cumulative mortality up to 7 days after treatments in the airgun exposed *C. finmarchicus* (GLM, n_bags_=4 (per treatment), *p* = 0.63). On day 7, the mortality in the airgun exposed copepods was 13.3 ± 5.8% in comparison to 8.8 ± 6.2% in the control.

## Discussion

The results of this study showed that discharge from seismic airguns (a maximum of 182 dB re 1 µPa^2^ s) caused no detectable change in *in situ* zooplankton immediate mortality or vertical distribution (center of mass). However, zooplankton spread (inertia) increased closer to the seismic source, and the cultured copepods near the source exhibited increased mortality up to 7 days after exposure.

In our study, the immediate mortality of wild *Calanus* spp. surpasses that previously reported in *C. finmarchicus*^18^, where only two small airguns were used. On the other hand, the mortality was considerably lower (<30%) than the levels reported in McCauley et al. (2017) (~45%) who used lower sound levels (156 dB re 1 µPa² s^-1 ^from 509 to 658 m)^19^. They also found high mortality in samples primarily dominated by krill larvae and smaller copepods like *Acartia* spp. In contrast, Pearson et al. (1994)^25^ and Parry et al. (2002)^23^ found limited to no effects of airgun blasts on zooplankton, albeit with other species than those studied here. These disparities suggest that effects on zooplankton may be influenced by the number of airguns, directionality of the exposure or be species specific. For example, our study showed no immediate effects on *Calanus* spp. yet smaller copepod species or developmental stages might respond differently to seismic exposure. For example, Vereide et al. (2023)^24^ reported increased mortality and decreased development in response to seismic exposure on *Acartia tonsa* nauplii and Vereide et al. (2024)^20^ reported a higher mortality in *Acartia* than in *Calanus* when comparing the effect of a pressure drop.

Accounting for background mortality is essential for accurately assessing the effects of anthropogenic disturbances on zooplankton. In areas without seismic activities, natural mortalities range from 11.6 to 59.8% (reviewed by^29^), reflecting high natural non-predatory mortality due to factors like senescence, turbulence, temperature, or parasitism. The high variation in natural mortality mirrors the observations in our study, where the mortality varies along the entire seismic approach (Fig. 4). In fact, background noise levels from our research vessel (158 ± 0.5 dB re 1 µPa² s; broadband SEL) exceeded reported levels of airguns from similar studies^19^.

Our bag experiments provided a controlled setup eliminating sources of potential damage by vessels, e.g., propeller turbulence^30^. Overall, the exposure of *C. finmarchicus* in bags did not show a discernible pattern in immediate mortality, with consistent rates observed up to 6 km from the seismic source. Nonetheless, initial sub-lethal or delayed effects may still be present, as indicated by the delayed mortality in the copepods that were exposed to the seismic source nearby. Although not measured in this study for *in situ* zooplankton, delayed effects are a critical aspect of the overall impact and should be considered when assessing the broader implications. It is also important to note that increased predation, caused by e.g., physical harm or less activity, is excluded when using bags. Therefore, additional indirect causes of mortality directly due to the airguns should be considered in future investigations.

The biomass distribution showed that zooplankton congregating around the pycnocline and subsurface chlorophyll maximum, as is often the case^31–34^. While the center of mass (CM) of the zooplankton layer remained constant with distance from the seismic source, the spread (inertia) varied along the transect, indicating shifts in the distribution of individuals within the layer. This suggests that certain taxa might be more affected than others or move asynchronously within the layer^19^.

While both copepods and echinoderm larvae are common in similar areas^35^, the community composition may be influenced by offshore constructions and activities. For example, the installation of wind turbine foundations can create new hard substrates, potentially leading to an increase in the population of hard-bottom species and thereby also meroplankton^36^. Possibly, the large presence of echinoderm larvae may be partially attributed to offshore constructions, fostering alterations in the local food web dynamics^37^.

In conclusion, the data show limited direct impacts of seismic activity on zooplankton mortality and distribution, and a potential for a delayed impact due to delayed mortality. The natural variation in mortality and vertical distribution exceeded the effect of seismic exposure on *in situ* zooplankton, indicating that direct effects of seismic surveys on zooplankton are limited and species-specific. Whether the delayed effect on mortality we found can lead to population-level impacts needs to be further investigated.

## Materials and methods

**Fig. 6:**
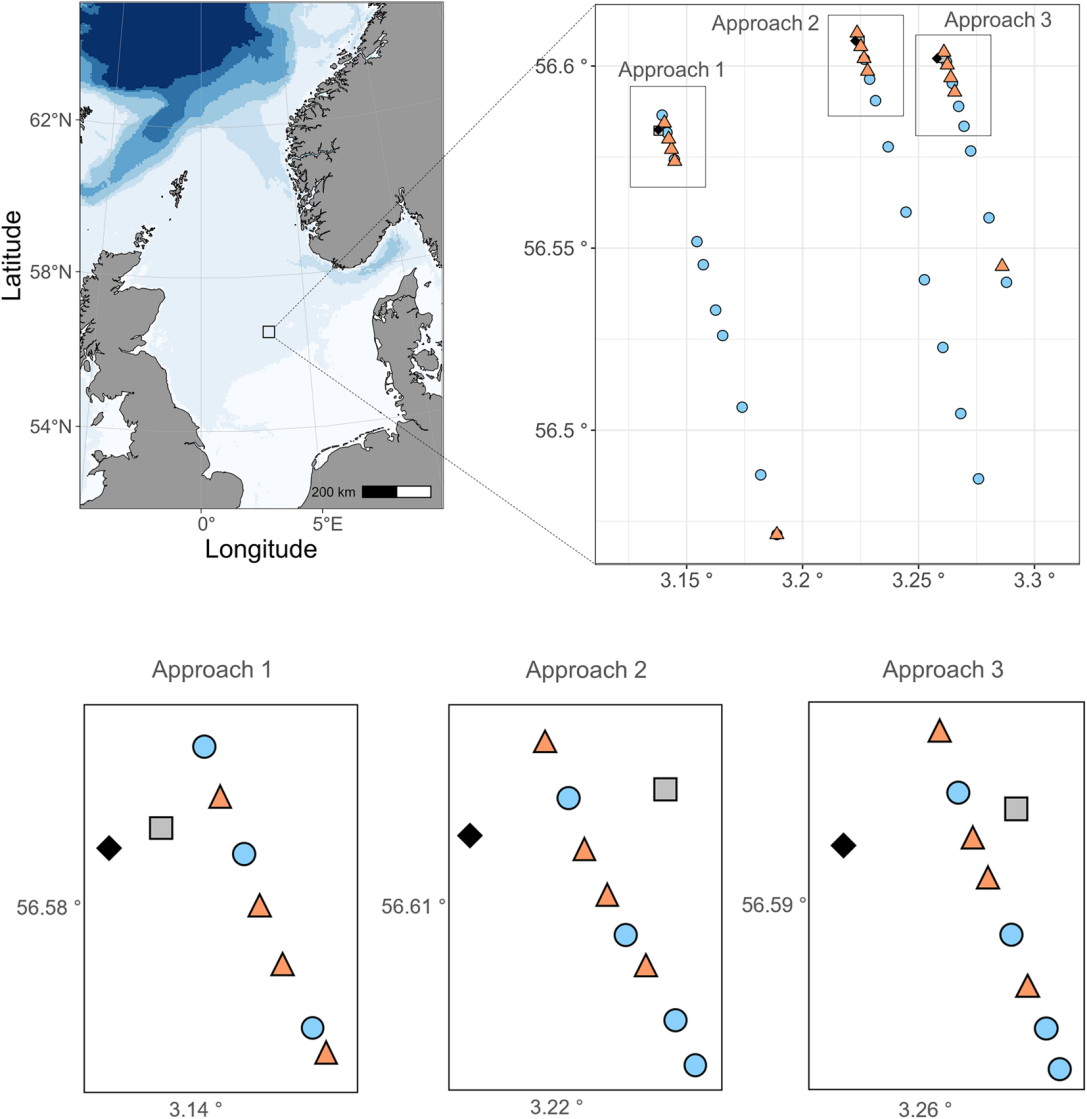
Map of the study site and overview of the positions of the two vessels during experimental stations. Left upper panel) location of the survey area in the North Sea. Right upper panel) Overview of the position of the stationary research vessel (grey square), the WBAT (bottom mounted echosounders) (black diamond) and the position of the approaching seismic vessel when WP2 plankton net (blue circle) or line with bags (red triangle) were deployed by the research vessel (in the grey square position). The three panels show seismic approaches 1, 2 and 3. Lower panels) Close-up view of each of the seismic approaches near the WBAT and the research vessel. The three seismic approaches were conducted 1st of May (approach 1), 2nd of May (approach 2), and 5th of May (approach 3).

This study was conducted in the North Sea (3°20 E, 56°55 N; Fig. 6), between the 1st and 5th of May 2022. The experiments (Table 1) were carried out aboard the Institute of Marine Research (IMR) research vessel RV Kristine Bonnevie during a seismic survey, where a seismic airgun array surveyed along parallel lines. To examine zooplankton layers acoustically before, during and after an approach of a seismic vessel, bottom-mounted upward-looking Wide Band Autonomous Transceivers (WBAT; EK80), were located at the end of three of the seismic lines. Prior to each seismic line being surveyed, the research vessel was positioned at the end of the seismic line, where net sampling and exposure experiments were conducted to assess zooplankton mortality at varying distances from the seismic source. The seismic vessel then approached the stationary research vessel and echosounder at 4.5 knots, firing along the seismic line. This procedure - referred to as seismic approaches 1–3 throughout the paper - was repeated three times. No seismic activity occurred within 2 nautical miles of the observation station for at least 48 h before each approach.

**Table 1:** Overview of all sampling days, at what days the seismic survey was running, days of seismic approach (1–3), and zooplankton field experiments of zooplankton mortality (WP2 plankton nets; immediate mortality, cultured *C. finmarchicus*; immediate and delayed mortality).

Date	Experiments conducted			
	Seismic survey running	Seismic approach	Immediate mortality: WP2 plankton nets [stain conc. (ml stock/L), time (min)]	Immediate/ delayed mortality: Cultured C. finmarchicus
01.05.22	Yes	Approach #1	Yes [0.3/1, 15]	Exposed, handling control
02.05.22	Yes	Approach #2	Yes [0.3/1, 15]	Exposed, handling control
03.05.22	No	–	–	Control
04.05.22	Yes	–	–	–
05.05.22	Yes	Approach #3	Yes [1.5/1, 15]	Exposed, handling control

At the observation site, multiple parameters were measured before, during, and after each seismic approach. Environmental conditions were assessed using CTD casts, along with measurements of chlorophyll-a, turbidity, and light intensity. Sound levels were recorded at 10 m depth with a hydrophone during each seismic approach. Zooplankton species composition at discrete depths was sampled using Multinets before and after each approach. Continuous WP2 net tows from the research vessel allowed for immediate mortality assessment using Neutral Red Stain. Finally, to assess the effects of the seismic sound in the absence of propeller damage or possible previous seismic exposure, cultured *C. finmarchicus* (CV/CVI stages) were exposed to seismic airguns in bags placed at various distances and times during each seismic approach. Detailed descriptions of each experimental method are provided below.

### Study site

The field experiments took place in the southern part of the Norwegian sector in the North Sea (3°20 E, 56°55 N) across a uniform depth of ~70 m (Fig. 6). Seismic surveys have been conducted annually in this area since 1971. In 2022, the seismic survey began on 18th of April, 13 days before the field experiments.

To ensure that the zooplankton drifted in the direction of the research vessel after exposure, the research vessel was positioned downstream from the approaching seismic vessel. To guide this positioning, Copernicus Marine Data Store provided predictions from the Northwest Shelf (NWS) oceanographic model. A custom Python script, utilizing the MOTU APU, was employed to access and download daily predicted speeds and directions in the specific area of experiments. A comparison between model predictions and measurements from a vessel-mounted ADCP (RDI 150 kHz ADCP) was conducted.

### Environmental data

The conductivity, temperature, and depth were measured with a CTD (SBE 911plus). The CTD was equipped with a fluorometer (Chelsea Aqua 3), transmissometer (LI-COR Biospherical), and oxygen sensor (SBE 43). Chlorophyll concentrations were measured in water samples collected with NISKIN bottles (5 L) at different depths (~5, ~10, ~20, ~30, ~50, and ~65 m). The water samples were filtered, the filters extracted in 90% acetone, and the extracts centrifuged and measured fluorometrically using Turner Design model 10 AU^38^.

### Seismic airgun source and survey operations

An airgun array of 24 (12 clusters × 2 airguns) Sercel G-Gun II airguns (operating pressure 2000 psi, total volume 3060 inch^3^) was used as the seismic source during an ongoing seismic survey. The array consists of three sub-arrays (6 m apart) with a length of 8.55 m and 12 m width. The depth of the airguns was 5 m ± 0.5 m below the surface. The airgun source was towed 50 m behind the seismic vessel and discharged every 50 m (every ~10.7 s), while the vessel traveled at 4.5 knots.

### Sound measurements

Ambient sound and the sound from the seismic survey was recorded with an Ocean Sonics Eth-X2 hydrophone (sensitivity 205 dB re 1 µPa) deployed at 10 m depth midship at the port side of the research vessel. The hydrophone recorded at varying time intervals during each of the three seismic approaches. During the approaches, the seismic ship was approaching from ~10 km until it passed the research vessel, whereupon shooting stopped at the end of a seismic line. In addition, we recorded ambient sound without seismic shooting.

In our acoustic analysis, we measured the broadband Sound Exposure Level (SEL) across the frequency range of 10 Hz to 20 kHz over 10 s intervals. In addition, we reported the maximum peak-to-peak pressure (Pa) in each 10 s interval. Given that the intervals (10 s) used in the sound analyses were shorter than the actual airgun shot intervals (~10.7 s), some measurements did not include seismic shots.

### Zooplankton vertical movement

#### Bottom-mounted echosounders (WBAT)

Three bottom-mounted upward-looking echosounders, WBAT EK80, were deployed at fixed positions prior to the cruise (12th of April 2022; Fig. 6). The positions were set at the end of the three seismic lines (Fig. 6). Each WBAT continuously recorded acoustic data from 5 m above the seafloor at 200 kHz.

To assess the distribution of zooplankton, we utilized the center of mass and inertia as our primary indicators^39^. The center of mass (CM), determined through volume backscattering coefficient values (*sv*; dB re 1 m^− 1^), served as key metrics for depicting the spatial arrangement and variability of population densities^40^. For the associated equations, see Supplementary Material. The inertia (I) represented the dispersion (spread) of zooplankton along the water column^39^. Active acoustic data was analyzed using LSSS (Large Scale Survey System, version 2.14.0) software. The surface layer (depth varying between 5 and 20 m depending on weather conditions) was excluded from the analysis due to its susceptibility to disturbances from the surface (e.g., wind, turbulence). The echogram analysis involved distinguishing acoustic signals from different animals, focusing particularly on backscatter from zooplankton^41–43^. Reports were generated in the form of nautical area scattering coefficients (s_A_) gridded with a resolution of 2 m on the depth-axis and 2 s on the time-axis of the echogram.

To select the appropriate frequency for zooplankton detection, factors such as size, shape, and density must be considered. While 333 kHz would likely provide better resolution for copepods, particularly *Calanus* species^42^, it is limited to a range of about 50 m, whereas 200 kHz extends to 100–200 m, covering a larger portion of the water column. In this study, all three WBATs offered 200 kHz, while only one supported 333 kHz. We therefore used the 200 kHz in our analyses and used the 333 kHz to confirm that the 200 kHz reflected mainly zooplankton (Supplementary Material).

#### Multinets

Zooplankton vertical distribution was examined with a 0.25 m^2^ HydroBios Multinet (180 μm mesh size) and sampled at discrete depth intervals during a vertical haul (60 − 45, 45 − 30, 30 − 20, 20 − 10, 10 – 0 m). Each haul was conducted at a mean retrieval speed of 0.176 m^− s^. The Multinet was deployed at the beginning (4–6 km from the seismic vessel) and end of each seismic approach (~1 h after passing). In addition, one Multinet was deployed as a night control (3rd of May, 21:19 UTC). The Multinet zooplankton samples from each depth interval were split using a Motodo plankton splitter and half of the final sample was further split into three size fractions (<2 mm, 1–2 mm, 1–180 μm), rinsed in freshwater, and then dried. From the 2 mm fraction, chaetognaths, fish larvae, krill, and amphipods were identified, enumerated, and dried. The remaining half of the final sample was fixed in 4% borax-buffered formalin for later identification and counting. A complete overview of species distribution is provided in the Supplementary Material. The Multinet samples were also used for ground truthing the acoustic data.

### Zooplankton mortality

#### Immediate mortality of field caught zooplankton

To determine the immediate mortality of *in situ* zooplankton, WP2 nets (180 μm mesh size) were deployed multiple times as the seismic vessel approached (Fig. 6). This procedure was replicated three times (seismic approaches 1–3). The net was hauled from near the bottom (~2 m) to the surface. We used WP2 nets instead of Multinets, for this purpose, because these allowed for a higher sampling frequency as the seismic vessel approached.

*Staining*

To quantify the proportion of live and dead copepods, the samples from the WP2 nets were stained with Neutral Red Stain (Table 1), following Elliot and Tang (2009)^44^. After rinsing, the samples were split, and ½ were preserved in formalin (4% buffered), and the other ½ split again. The two ¼ fractions were photographed in Petri dishes using a Sony α7R IV 35 mm full-frame camera (ILCE-7RM4) equipped with a Sony FE 90 mm f/2,8 Macro G OSS lens with manually fixed settings. All images included a scale bar for size calibration. Light was provided from below by a Reflecta light Table (10319), and the entire setup was covered by a black tent. The images were stored as 60.1 Mpixel raw files. Finally, the samples were filtered through a 100 μm mesh (⌀=4.5 cm) and stored in Petri dishes (⌀=5 cm) at -20 °C.

At all staining times, we also stained, photographed, and stored batches of dead zooplankton to control for the diffusive uptake of pigment in dead zooplankton^44^. As the animals in our study appeared lighter than the images in Elliot and Tang (2009)^44^ and Daase and Søreide (2021)^45^, we conducted an ad hoc test of the effect of staining time. While increased staining time may facilitate visual inspection, our automated image analysis correctly classified animals as alive or dead at all staining times.

*Image analysis*

To ensure objectivity within the subtle staining gradients, we developed macros within the open-source image analysis software ImageJ (version 1.53e;^46^). These macros enabled the application of color thresholding to highlight areas that had reached specific staining levels. Pixels meeting these criteria were marked in a vivid green color. We then analyzed color-thresholded control images of live and dead plankton samples. Plankton displaying green marking, as determined through visual observation, were classified as live (see Supplementary Material for examples). Our images were then systematically categorized into taxa and dead/live using an ObjectJ macro that allowed manual counting and marking into categories.

#### Immediate and delayed zooplankton mortality—cultured *C. finmarchicus*

To assess delayed and immediate mortality, we used cultured *C. finmarchicus* of known origin and exposure history. The animals were sourced from the running culture at NTNU SeaLab in Trondheim^47^. Prior to departure, approximately 3000 copepodites in stage V were shipped as air freight in insulated containers at a density of 75 individuals L^− 1^. *C. finmarchicus* (CV) was maintained at 10 °C in a climate container on deck on a light: dark cycle of 12:12 h, in 20 L buckets (375 individuals in each bucket) filled with 0.2 μm filtered seawater (FSW) with continuous aeration.

*Pre-exposure*

Before each experiment, 10 or 50 individuals (Table 2) were transferred to transparent plastic bags (Lamizip^®^, 1 L, internal measures 144 × 227 mm, thickness 0.16 mm, PET/PE) filled with FSW (10 °C). We enclosed the bags with no air inside. The bags were attached to a set of lines, in random order but at a given distance (Table 2). Bags with 10 individuals were placed 0.8 m apart with three bags per line (Table 2). Bags with 50 individuals had 0.4–0.8 m between bags with four bags per line.

**Table 2:** Overview of all zooplankton bags; date, treatment of the cultured *C. finmarchicus*, and an overview of number of lines per seismic approach, bags per line, animals per bag, and duration of exposure.

Date	Treatment	# Lines/ seismic approach	# bags/ line	# animals/ bag	Time total in water/ at 10 m depth	Description
30.04.22	Control (3 nm)	4	3	10	45 s/ 15 s	
01.05.22	Seismic approach #1	5	3	10	45 s/ 15 s	1 line at > 6 km, 4 lines at 89–1192 m
01.05.22	Handling control	1	3	10	–	Stayed in container
02.05.22	Seismic approach #2	5	3	10	45 s/ 15 s	1 line at > 6 km, 4 lines at 89–1192 m
02.05.22	Handling control	1	3	10	-	Stayed in container
03.05.22	Control	4	3	10	45 s/ 15 s	
05.05.22	Seismic approach #3	5	3	10	45 s/ 15 s	1 line at > 6 km, 4 lines at 89–1192 m
05.05.22	Handling control	1	3	10	-	Stayed in container
05.05.22	Seismic approach #3	1	4	50	45 s/ 15 s	1 line at 89–178 m
05.05.22	Handling control	1	4	50	-	Stayed in container

*Exposure*

The bags containing copepods (Table 2) were lowered into the water by a crane (at the same speed for all treatments, ~0.3 m^− s^) to a depth of 10 m (based on the middle bag). The bags were maintained at 10 m for 15 s before they were lifted back into the vessel. The 15 s window was chosen to allow multiple exposures to the seismic source from varying distances, rather than a single prolonged exposure.

With the bags of 10 individuals, this procedure was conducted with five sets of lines for each seismic approach (1–3). The first line of bags was deployed at the beginning of each seismic approach when the seismic vessel was 6–12 km away from the research vessel (dependent on the distance of the seismic line; Fig. 6). Thereafter, four lines of bags were deployed when the seismic vessel was near the research vessel (Fig. 6). Once the first line was onboard, we deployed the second, third, and fourth lines of bags, following the same procedure (Fig. 6). In addition to the three seismic approaches, there was a control treatment in which the same procedure was conducted without any seismic shooting (Table 2). We also kept three bags of 10 individuals in the climate container during exposure to control for mortality due to handling (handling control). With the four bags of 50 individuals, the procedure was only conducted at the closest distance to the seismic vessel (Table 2). Here, four additional bags were kept in the climate container for handling control.

*Post-exposure*.

Upon retrieval to deck, the bags were immediately placed in a climate container at 10 °C. We examined all individuals by visual inspection and noted whether they were dead or alive. Live copepods were transferred to individual bottles (50 mL, cell culture flask) prefilled with FSW (10 °C). Bottles were kept at 10 °C for 7 days, during which they were examined by visual inspection daily for mortality. Animals were not fed during the study period.

### Data analysis

All data analyses were conducted in the R statistical software (version 4.2.2;^48^). For all analyses, a significance threshold of 5% was used.

The effect of the approaching seismic airgun array on the vertical distribution of zooplankton was assessed using a General Linear Model (GLM) separately for each seismic approach. Due to differences in transducer configurations, the WBATs collected varying amounts of data. WBAT 2, equipped with two split-beam transducers, had to transmit sequentially on each channel, resulting in less than half the data collected on the 200 kHz channel compared to WBAT 1 (one split-beam and one single-beam) and WBAT 3 (one split-beam), which could transmit simultaneously. As seismic approach 2 (~ 5690 m) relied on WBAT 2 which was substantially shorter than approaches 1 (~ 7530 m) and 3 (~ 8820 m), and with fewer measurements, echosounder data analysis was limited to seismic approaches 1 and 3. In these models, the center of mass (CM) was used as the response variable and distance as the continuous variable. Similarly, inertia (I) was used as the response variable, with distance maintained as the continuous variable.

To test the effect of seismic exposure on the mortality of field*-*caught *Calanus* spp., we tested the correlation between proportion of dead individuals and distance from the seismic source using a Spearman correlation test. Additionally, we tested the difference in mortality between the *Calanus* spp. sampled during exposure and the *Calanus* spp. sampled after exposure using a Mann Whitney test.

We tested the effect of treatment on mortality in *C. finmarchicus* after exposure in bags in different ways. Firstly, to test the impact of treatment on cumulative mortality (up to 7 days after treatment) in the bags containing 10 individuals, we applied a binomial GLM. We used the proportion of dead individuals as a response variable. We included the treatment (seismic exposure close (89–1192 m), seismic exposure far (>6 km), control, handling control) and time after treatment (day 0 to 7) as fixed effects, as well as the interaction between time and treatment. Furthermore, to investigate the immediate mortality effects of treatment in the bags of 10 individuals, a separate binomial GLM was implemented. This model focused solely on mortality immediately after treatment and utilized the proportion of dead individuals as a response variable, and treatment as a fixed effect. Secondly, for the bags of 50 individuals, the effect of treatment on mortality was tested using a binomial general linear model (GLM). Similarly, we used the proportion of dead individuals as a response variable. We included treatment (seismic exposure (89–178 m from the seismic source) and handling control), and time after treatment (day 0 to 7) as fixed factors, as well as the interaction between time and treatment.

## Electronic supplementary material

Below is the link to the electronic supplementary material.

Supplementary Material 1

## Data Availability

The datasets generated during and/or analysed during the current study are available in the Amazon S3 service (Institute of Marine Research repository); this repository contains the raw data collected during the cruise. https://ftp.imr.no/nmdc/IMR/Doc/S3-Cruise.pdf.
