# Supplementary material for: Zooplankton mortality and distribution around a seismic survey
Source: Sci Rep. 2025 Sep 30;15:33907. doi: 10.1038/s41598-025-09465-2 (PMC12484595; doi:10.1038/s41598-025-09465-2)
Supplement: Supplementary file 1 — Supplementary Material 1 [file 41598_2025_9465_MOESM1_ESM.pdf]

Supplementary Information:

Zooplankton mortality and distribution around a seismic survey

Vereide, Emilie Hernes; Utne-Palm, Anne Christine; Titelman, Josefin; Pedersen, Geir; Strand, Espen; Mihaljevic, Marina; Kühn, Saskia; Altin, Dag; Thorsen, Anders; Campillo, Lucie; Fields, David M.; Khodabandeloo, Babak; de Jong, Karen

Supp. 1 Environmental conditions.

Water temperature in the upper 10 m ranged from 8.4 to 8.5°C throughout the study period, and below 40 m water depth, temperatures were stable at ~6.6°C down to the seabed. Salinity was in the range of 34.8-34.9 PSU. Environmental conditions were similar between approaches 1 and 2, but there was a windy period between approaches 2 and 3 in which seismic shooting was temporarily stopped. This windy period affected mixing and led to an increase in depth for the thermocline and the pycnocline, and increased light transmission during approach three.

The transmission data are reflected in the chlorophyll-*a* fluorescence. The chlorophyll-*a* concentrations peaked at ~5 m in seismic approach 1 (1.2 mg m^{-3}), ~20 m in approach 2 (1.1 mg m^{-3}), and ~30 m in approach 3 (0.7 mg m^{-3}). The surface water layer mixing was also seen in the echograms spanning the study period.

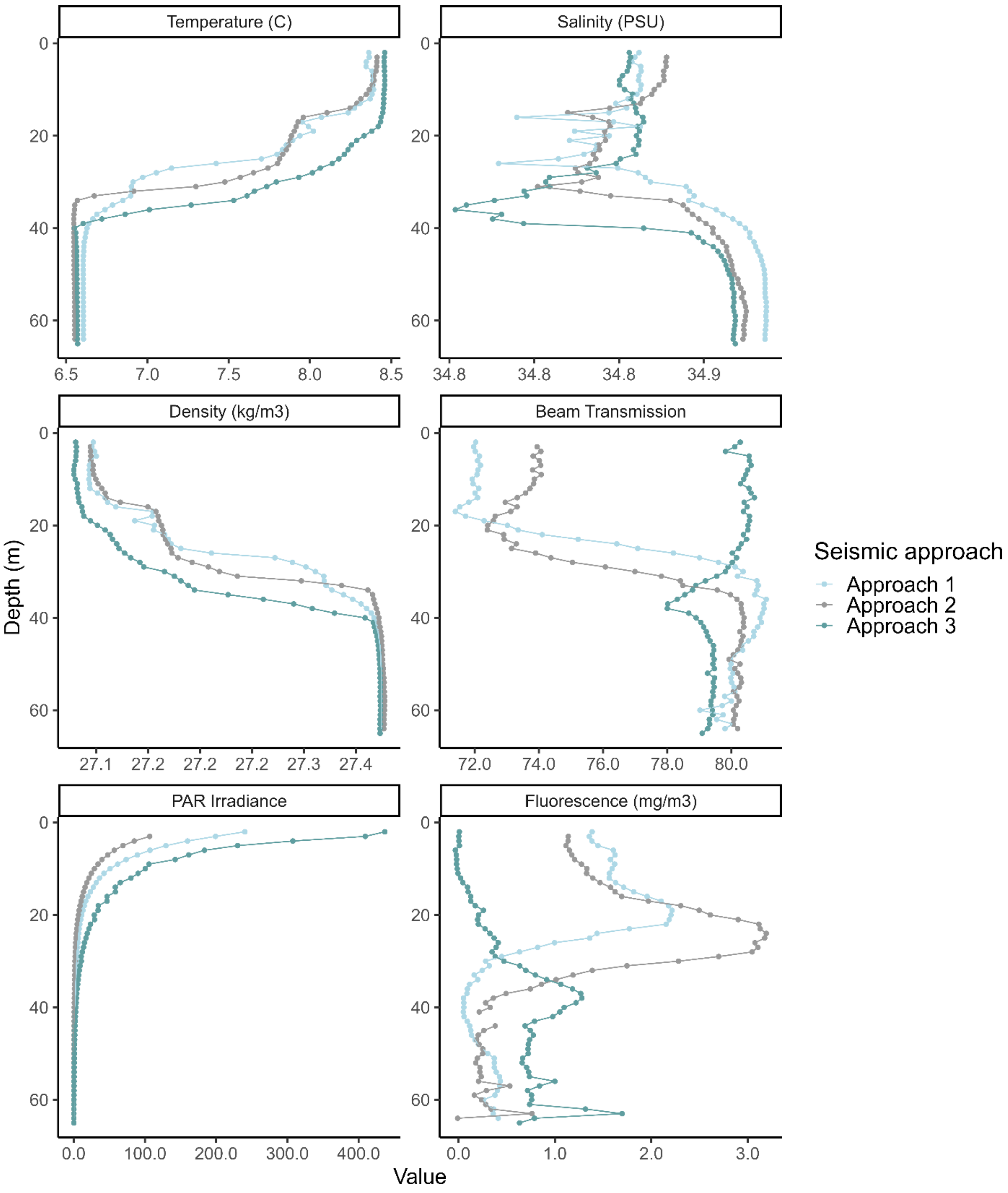

Supp. Fig. 1. The measured temperature (°C), salinity (PSU), density (kg m^{-3}), beam transmission (%), photosynthetically active radiation (PAR) irradiance (μmol/(m²·s)), and fluorescence (chlorophyll-*a*) (mg m^{-3}) for each of the three seismic approaches (1-3).

Supp. 2. Biological metrics used to summarize acoustic data.

The calculation of the center of mass (CM; m) is based on volume backscattering coefficient values (s_v) as detailed in the following equation (1).

$$(1)\ CM = \frac{\int z \times s_v(z)dz}{\int s_v(z)dz}$$

where z represents the depth (m); $s_v(z)$ the volume backscattering coefficient at depth (z) and dz signifies that we are integrating with respect to z.

Nautical area scattering coefficient values (s_A) were extracted from LSSS and converted into volume backscattering coefficient values (s_v) using the following equations:

$$(2)\ s_A = 4 \times \pi \times (1852)^2 \times s_a$$

$$(3)\ s_a = s_v \times d$$

where s_a is the area backscattering coefficient ($m^2.m^{-2}$), and d is the grid depth (m). Combining equations (2) and (3) yields:

$$(4)\ s_v = \frac{s_A}{(4 \times \pi \times (1852)^2 \times d)}$$

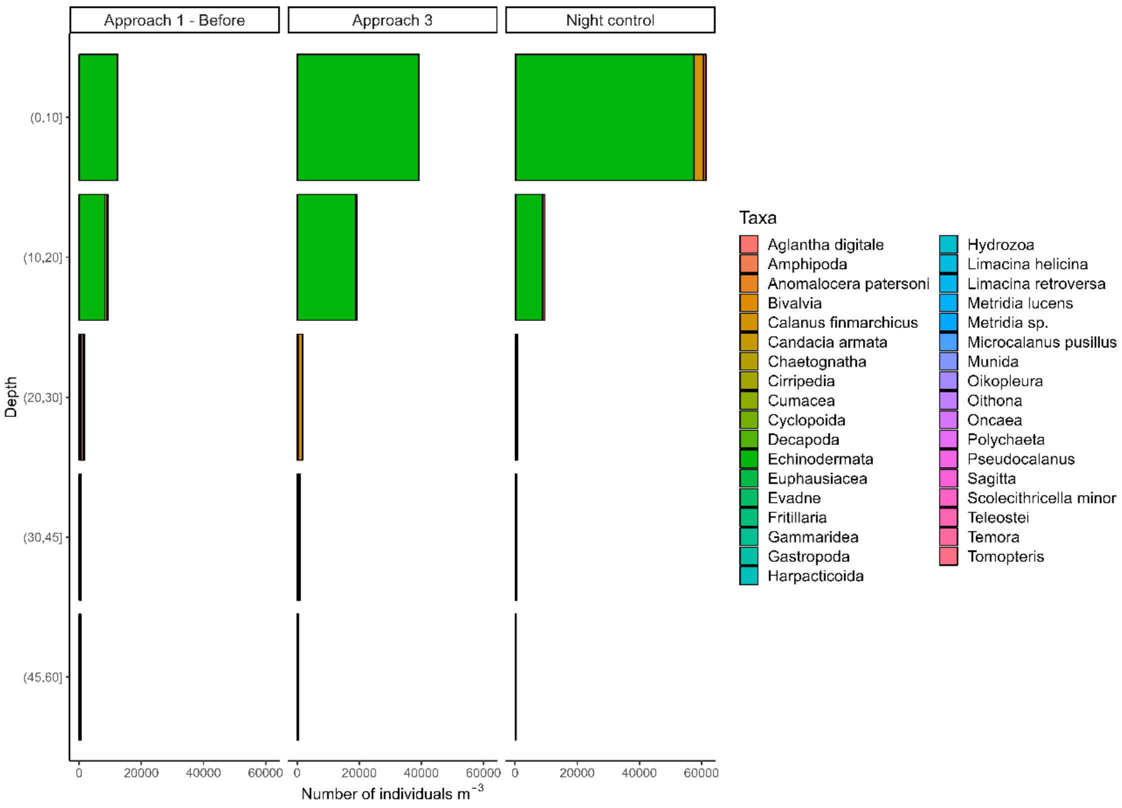

Approach 1 - Before
Approach 3
Night control
Depth
(0,10]
(10,20]
(20,30]
(30,45]
(45,60]
0
20000
40000
60000
Number of individuals m^-3
Taxa
Aglantha digitale
Amphipoda
Anomalocera patersoni
Bivalvia
Calanus finmarchicus
Candacia armata
Chaetognatha
Cirripedia
Cumacea
Cyclopoida
Decapoda
Echinodermata
Euphausiacea
Evadne
Fritillaria
Gammaridea
Gastropoda
Harpacticoida
Hydrozoa
Limacina helicina
Limacina retroversa
Metridia lucens
Metridia sp.
Microcalanus pusillus
Munida
Oikopleura
Oithona
Oncaea
Polychaeta
Pseudocalanus
Sagitta
Scolecithricella minor
Teleostei
Temora
Tomopteris

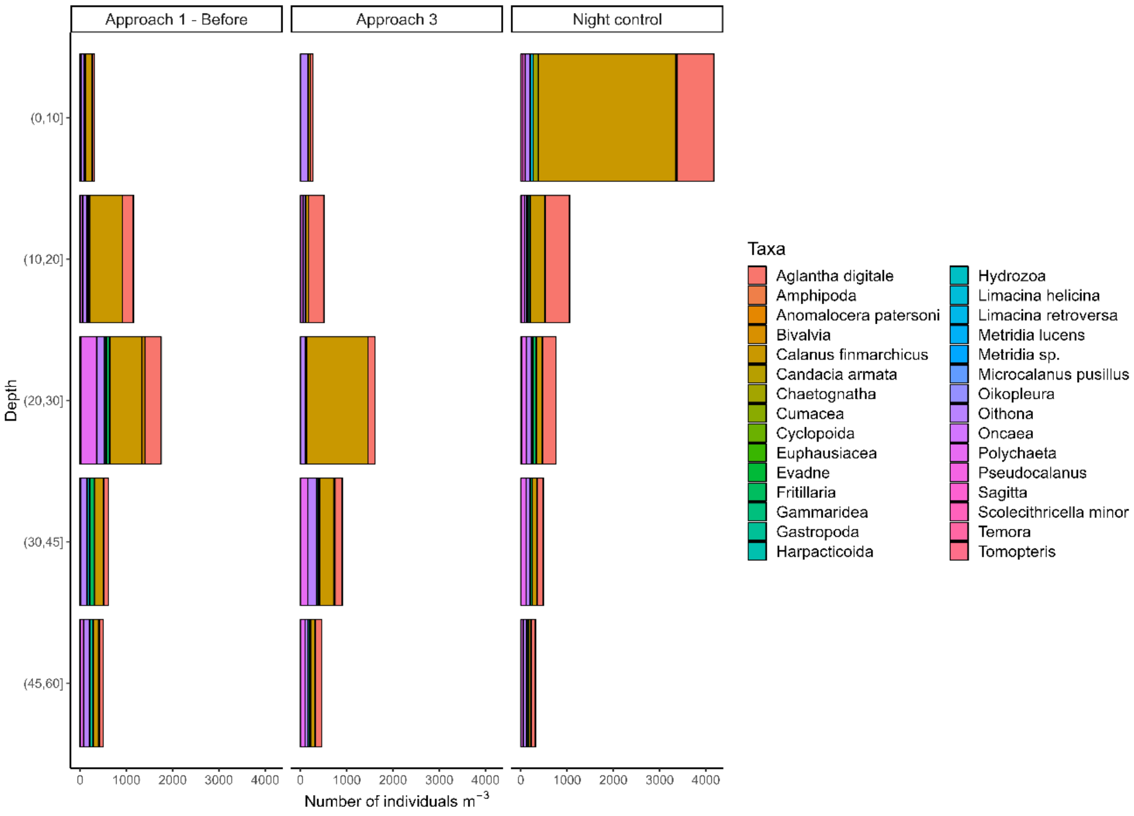

Approach 1 - Before
Approach 3
Night control
Depth
(0,10]
(10,20]
(20,30]
(30,45]
(45,60]
0
1000
2000
3000
4000
Number of individuals m^-3
Taxa
Aglantha digitale
Amphipoda
Anomalocera patersoni
Bivalvia
Calanus finmarchicus
Candacia armata
Chaetognatha
Cumacea
Cyclopoida
Euphausiacea
Evadne
Fritillaria
Gammaridea
Gastropoda
Harpacticoida
Hydrozoa
Limacina helicina
Limacina retroversa
Metridia lucens
Metridia sp.
Microcalanus pusillus
Oikopleura
Oithona
Oncaea
Polychaeta
Pseudocalanus
Sagitta
Scolecithricella minor
Temora
Tomopteris

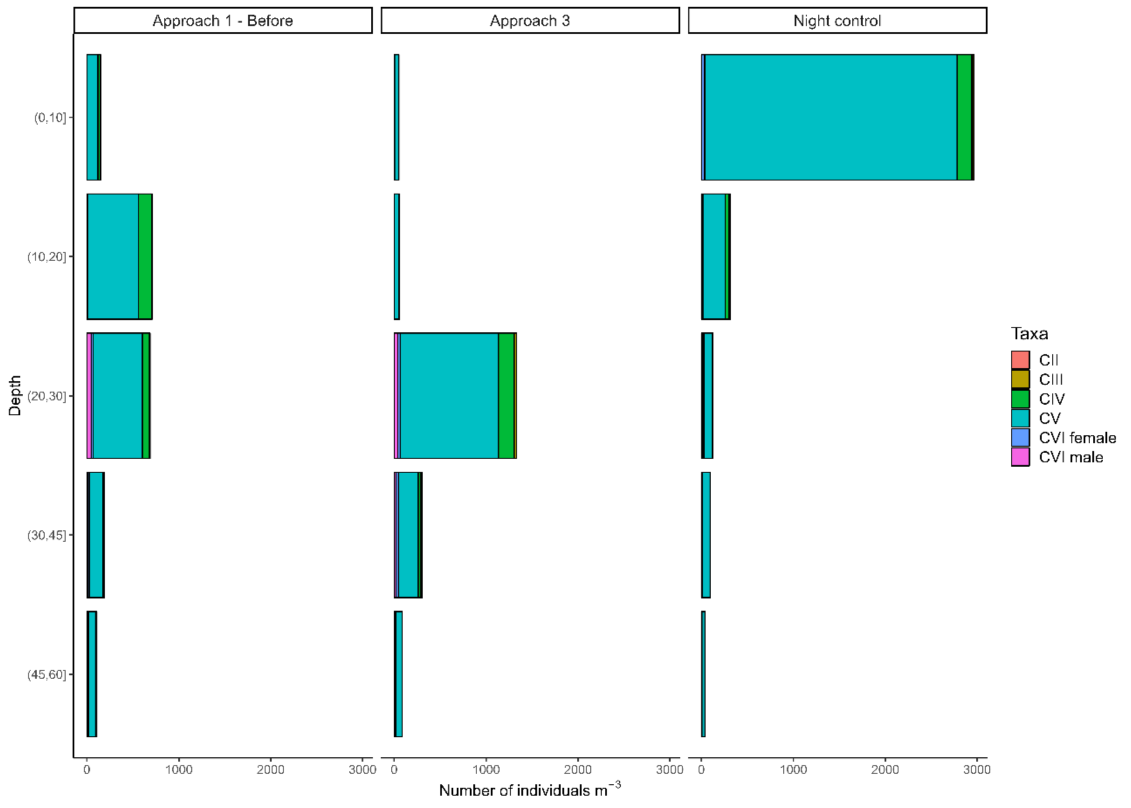

Supp. Fig. 2. Number of individuals (m^{-3}) for each depth interval (0-10, 10-20, 20-30, 30-45, 45-60) sampled before seismic approach 1, during seismic approach 3, and a night control. Upper panel) A complete overview of all sampled taxa, middle panel) overview of all taxa excluding echinoderm larvae (Echinodermata), and lower panel) overview of *Calanus* spp. developmental stages (CII-CV, CVI female and male).

Supp. Table 1. Complete overview of all taxa. Number of individuals (m^{-3}) sampled before seismic approach 1, a night control, and during seismic approach 3.

Station	Seismic approach 1: Before	Night control	Seismic approach 3
Species (per station)	573	578	579
Aglantha digitale	820	1558	945
Amphipoda		1	
Anomalocera patersoni		2	
Bivalvia	19	11	16
Calanus finmarchicus	618	593	581
Candacia armata	5	6	2
Chaetognatha	20	5	6
Cirripedia	2		
Cumacea		1	
Cyclopoida			1
Decapoda		3	
Echinodermata	1643	1931	1481
Euphausiacea			2
Evadne	2		
Fritillaria	41	61	20
Gammaridea		4	
Gastropoda	29	22	25
Harpacticoida	1		
Hydrozoa	81	4	2
Limacina helicina	1	3	3
Limacina retroversa	1		
Metridia lucens	11	17	9
Metridia sp.	2	3	2
Microcalanus pusillus	2		2
Munida	2		
Oikopleura	1		

Oithona	182	103	85
Oncaea	1	1	
Polychaeta	106	113	88
Pseudocalanus	11	8	7
Sagitta	20	67	15
Scolecithricella minor		1	1
Teleostei	11	6	3
Temora	3	12	1
Tomopteris		4	1

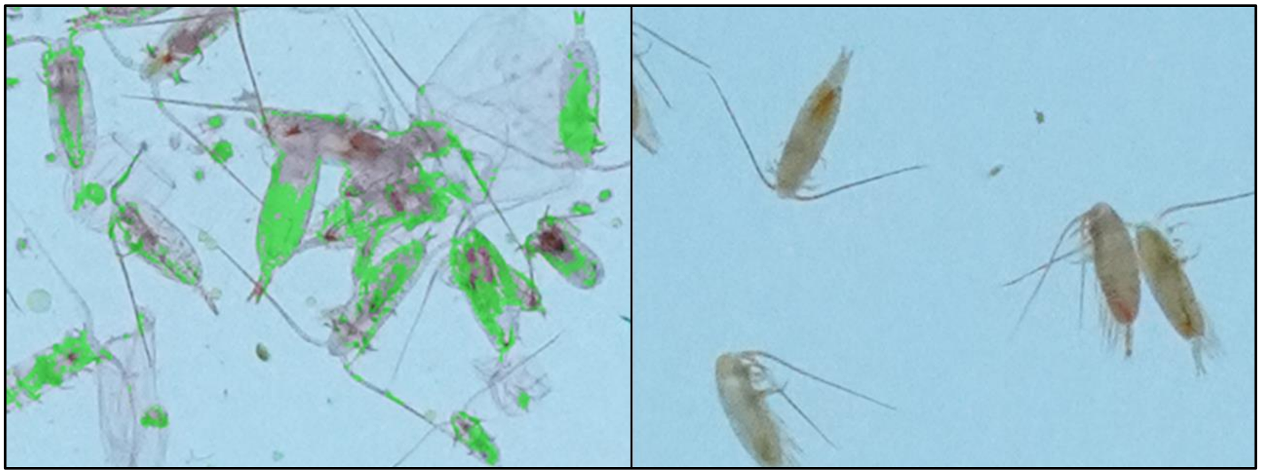

Supp. Fig. 3. Examples of stained animals live (left panel)/dead (right panel).

A) WBAT1 – Approach 2

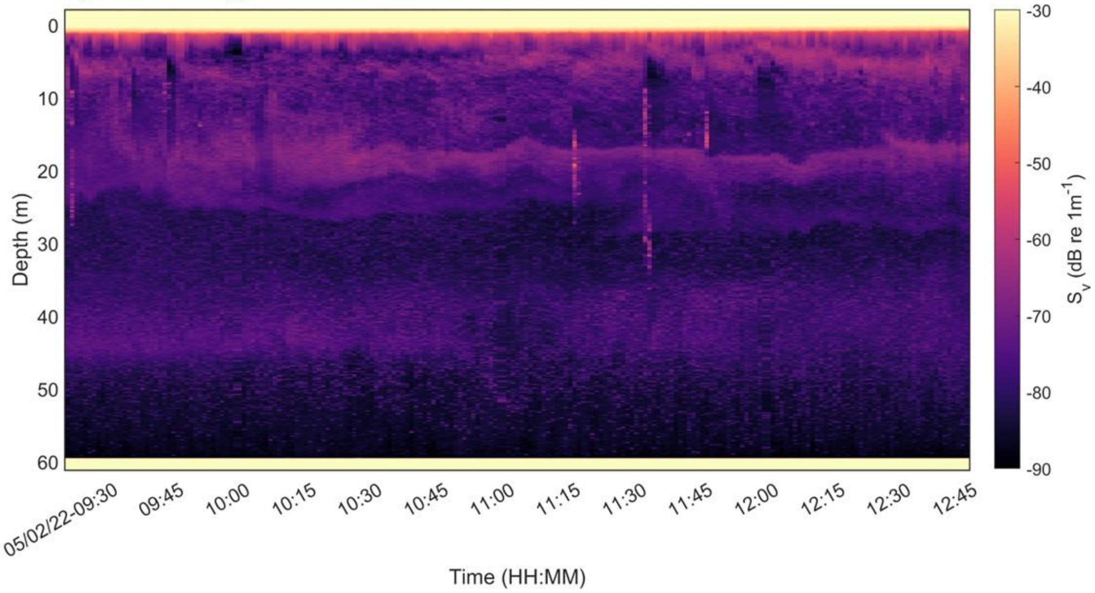

B) WBAT1 – Approach 3

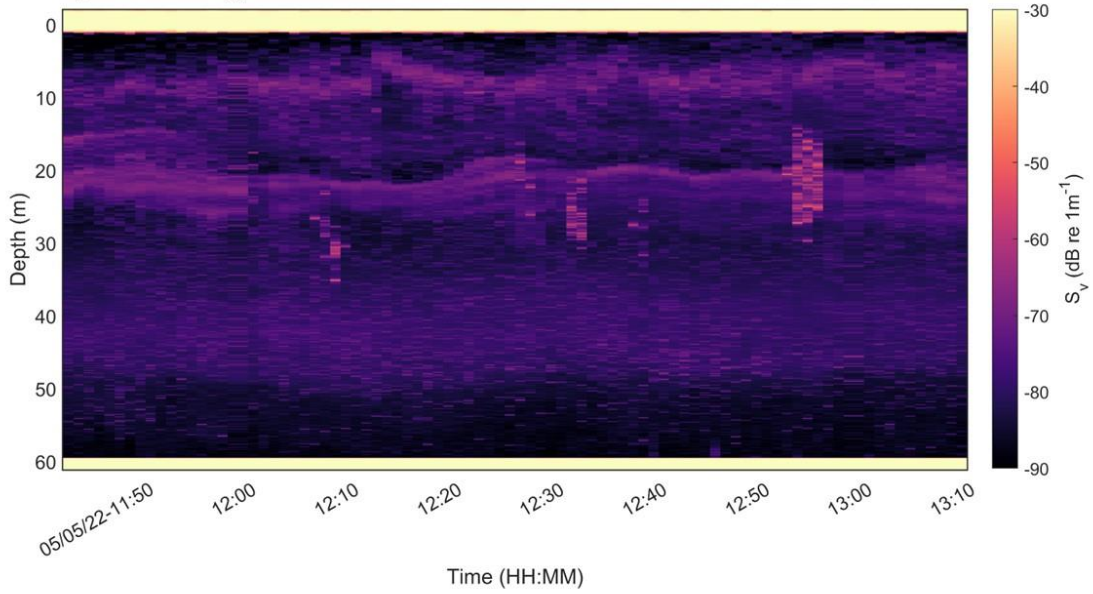

C) WBAT2 – Approach 1

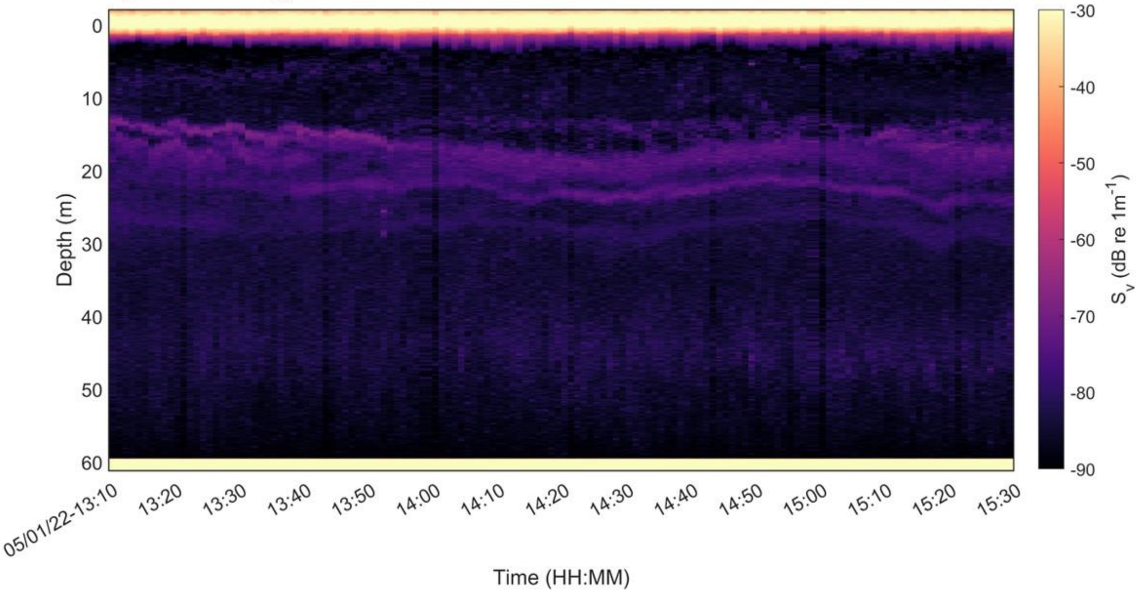

D) WBAT2 – Approach 2

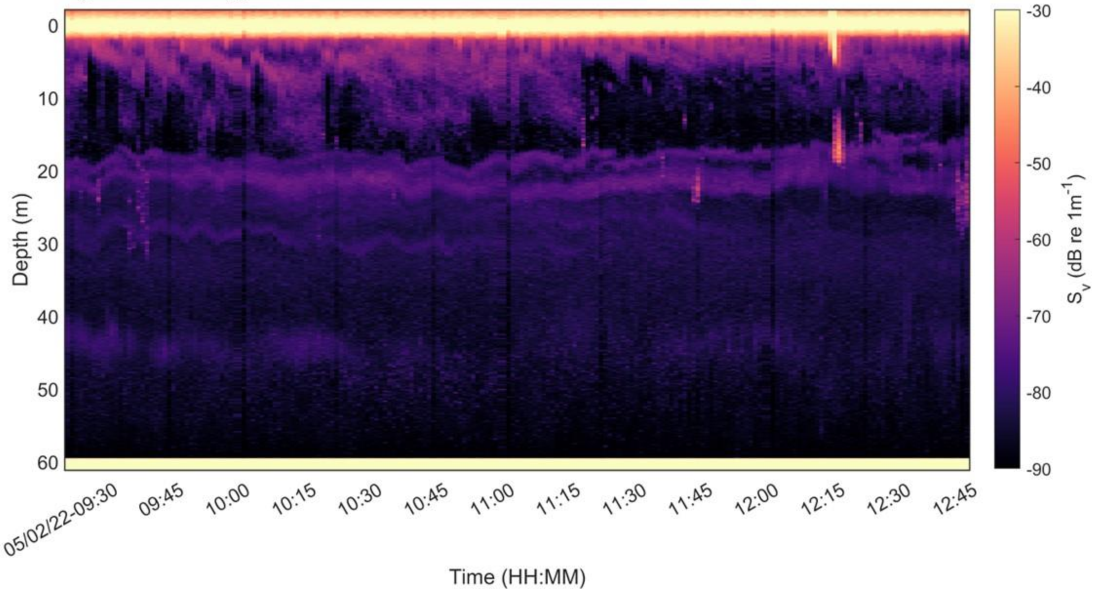

E) WBAT2 – Approach 3

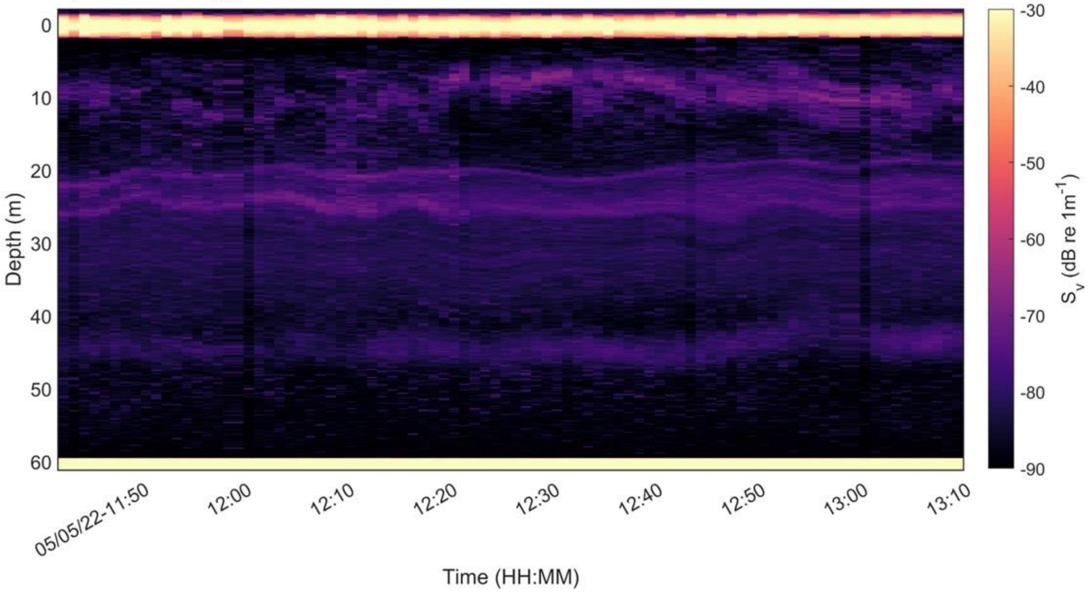

F) WBAT3 – Approach 1

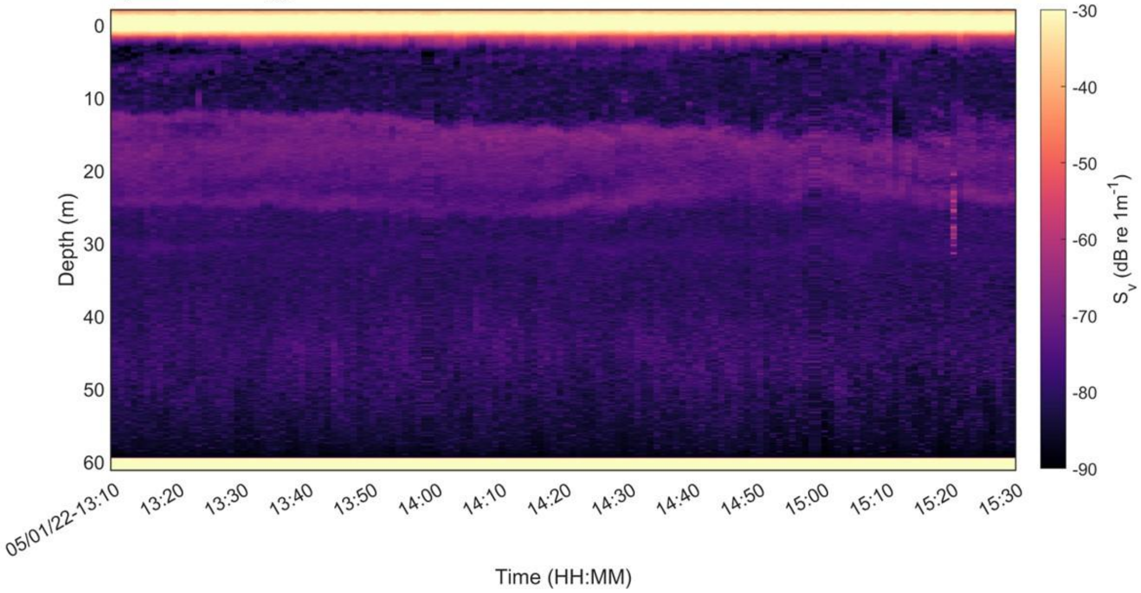

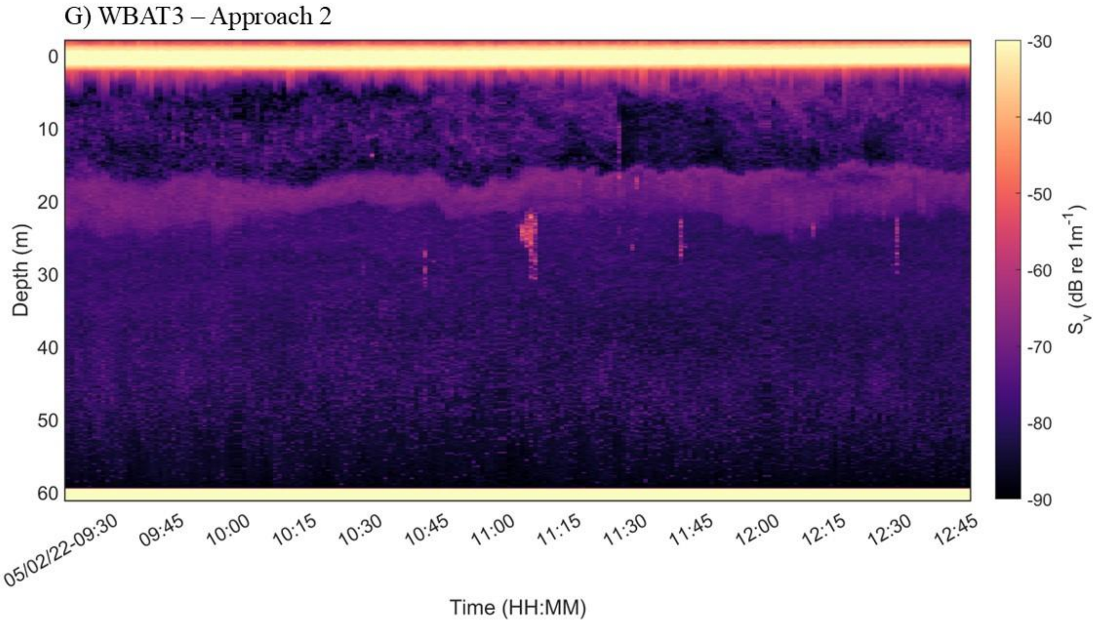

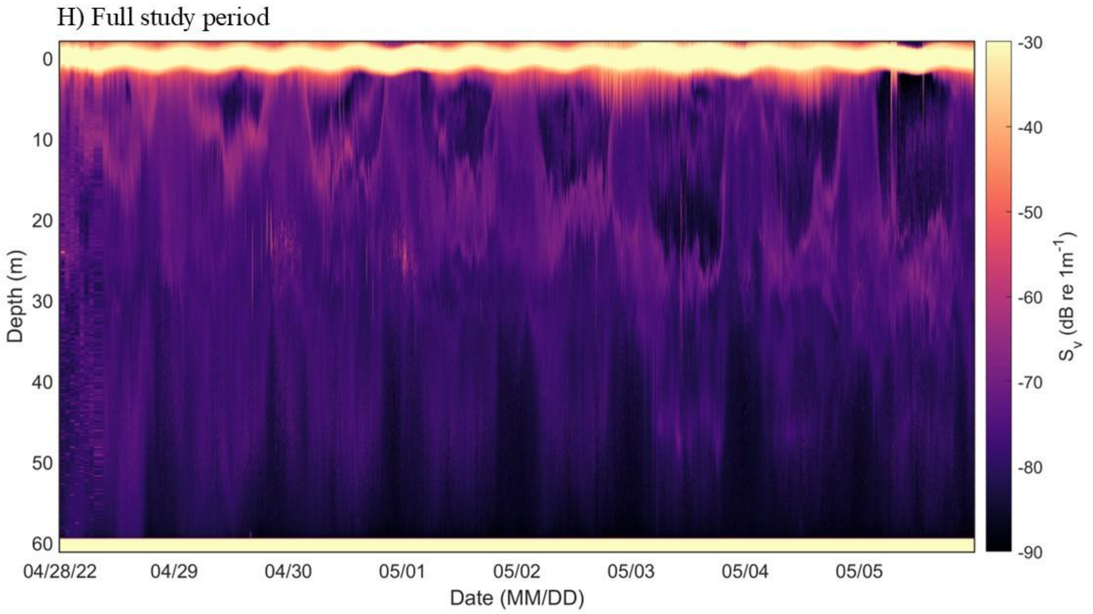

Supp. Fig. 4. Overview of the WBAT data from all seismic approaches during exposure.

A) WBAT1 – 200 kHz (01.05.22)

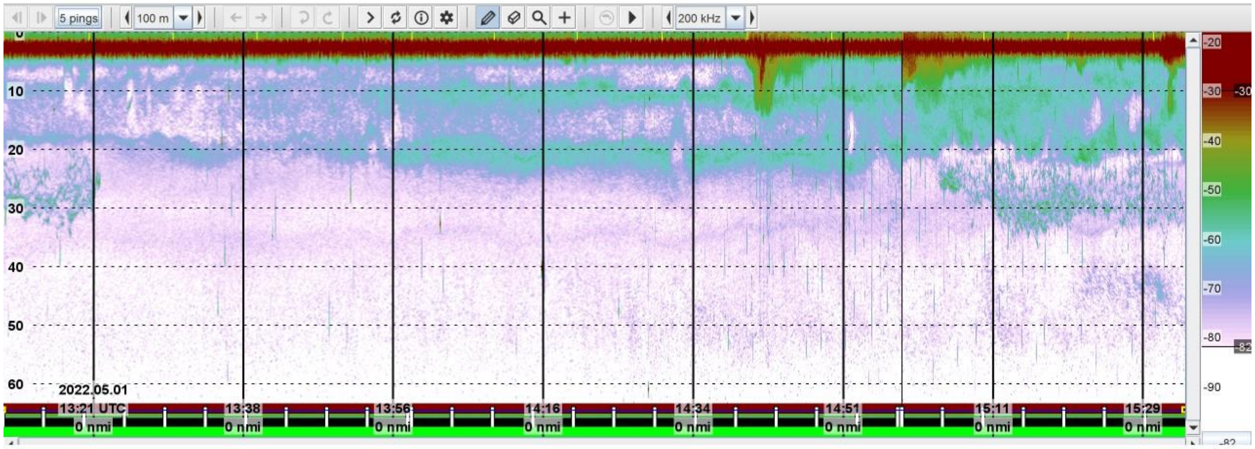

B) WBAT1 – 200 kHz (02.05.22)

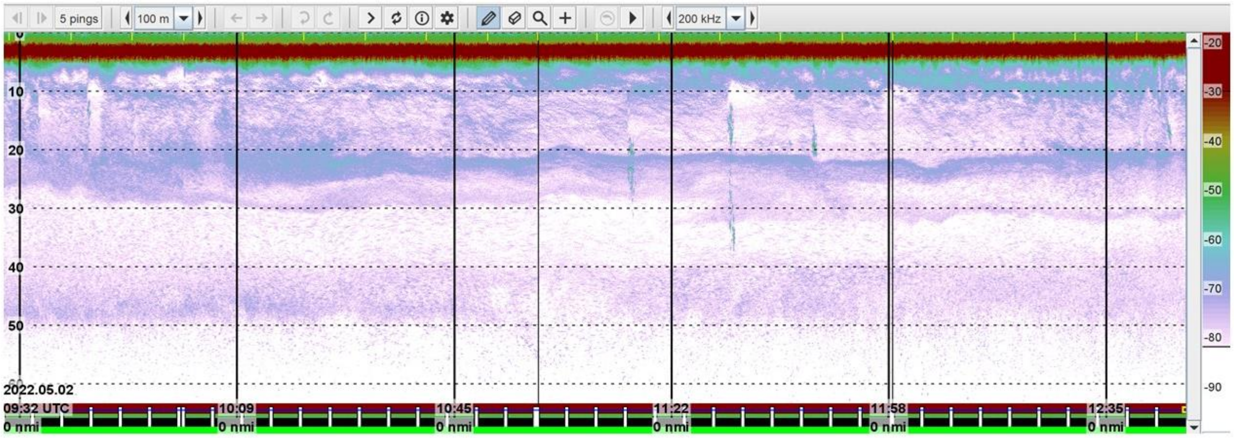

C) WBAT1 – 200 kHz (05.05.22)

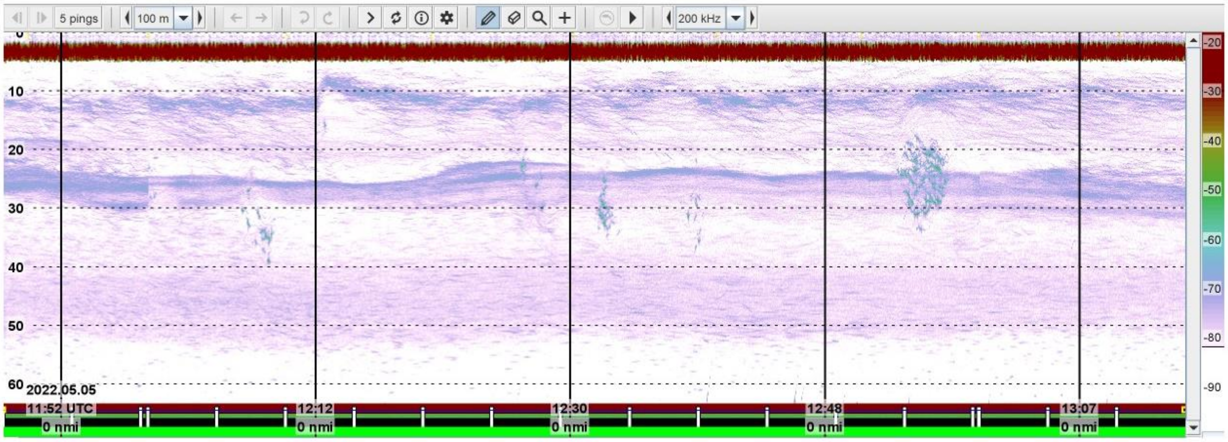

D) WBAT1 – 333 kHz (01.05.22)

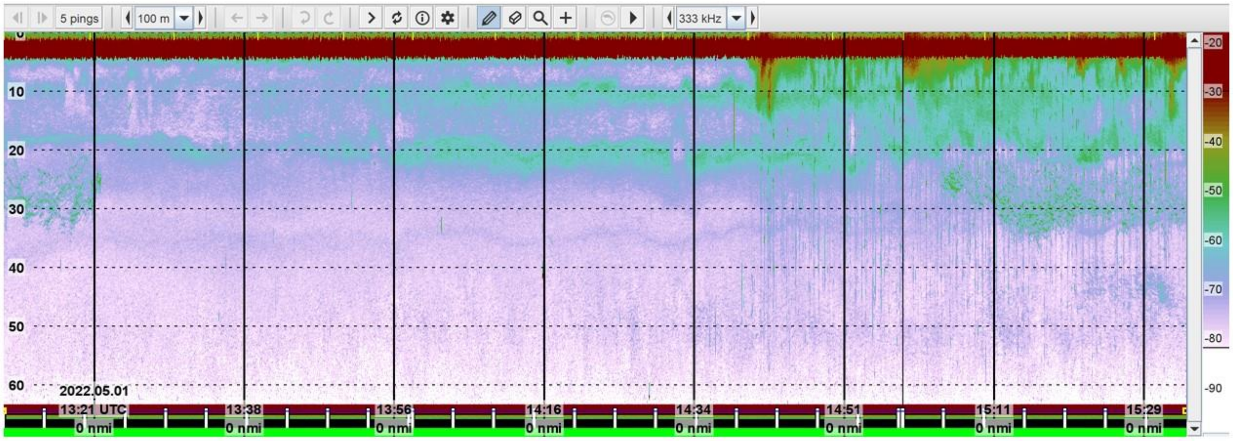

E) WBAT1 – 333 kHz (02.05.22)

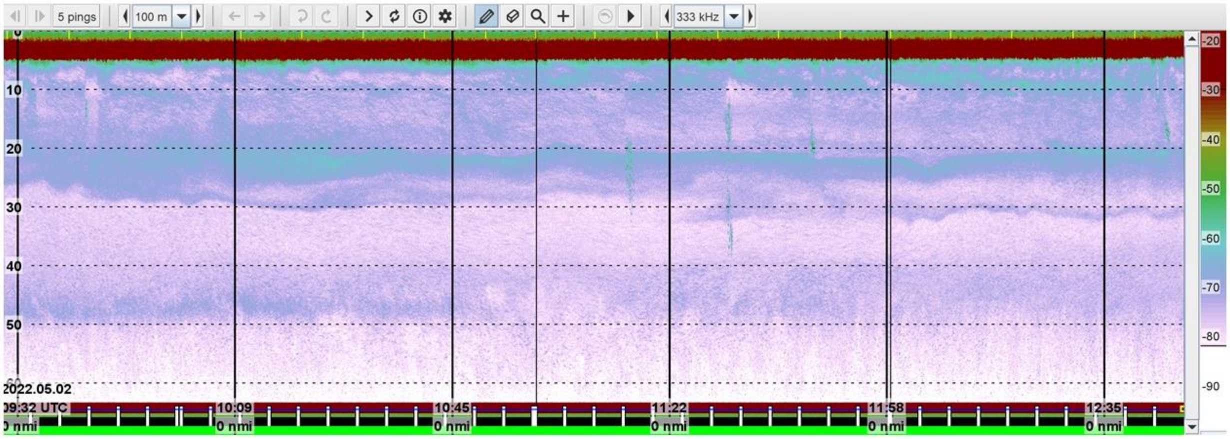

F) WBAT1 – 333 kHz (05.05.22)

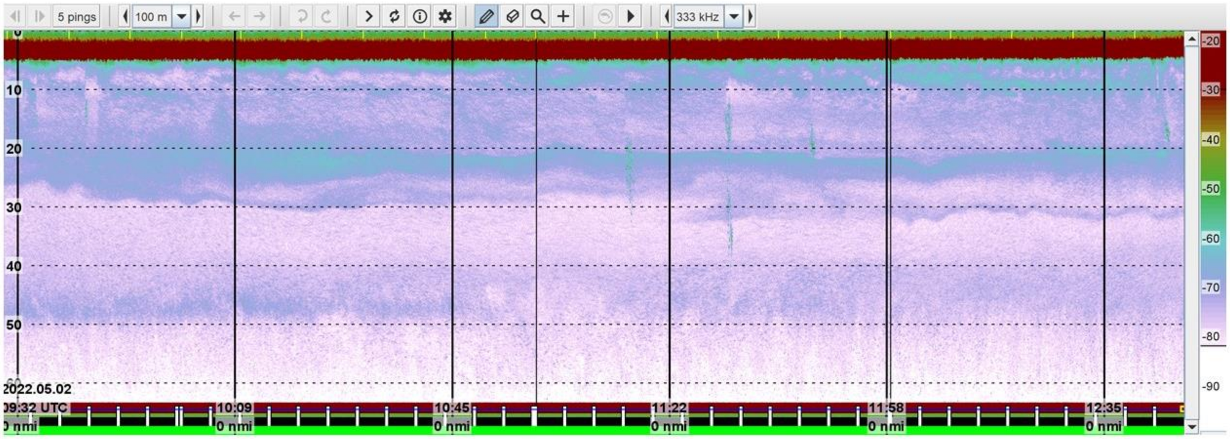

Supp. Fig. 5. Approach 1 to 3 during exposure demonstrating the use of both 200 kHz and 333 kHz.
